# Markerless Eye-Hand Kinematic Calibration on the iCub Humanoid Robot

**DOI:** 10.3389/frobt.2018.00046

**Published:** 2018-06-12

**Authors:** Pedro Vicente, Lorenzo Jamone, Alexandre Bernardino

**Affiliations:** ^1^Institute for Systems and Robotics, Instituto Superior Técnico, Universidade de Lisboa, Lisbon, Portugal; ^2^ARQ (Advanced Robotics at Queen Mary), School of Electronic Engineering and Computer Science, Queen Mary University of London, London, United Kingdom

**Keywords:** code:C++, humanoid robot, markerless, hand pose estimation, sequential monte carlo parameter estimation, kinematic calibration

## Abstract

Humanoid robots are resourceful platforms and can be used in diverse application scenarios. However, their high number of degrees of freedom (*i.e.*, moving arms, head and eyes) deteriorates the precision of eye-hand coordination. A good kinematic calibration is often difficult to achieve, due to several factors, *e.g.*, unmodeled deformations of the structure or backlash in the actuators. This is particularly challenging for very complex robots such as the iCub humanoid robot, which has 12 degrees of freedom and cable-driven actuation in the serial chain from the eyes to the hand. The exploitation of real-time robot sensing is of paramount importance to increase the accuracy of the coordination, for example, to realize precise grasping and manipulation tasks. In this code paper, we propose an online and markerless solution to the eye-hand kinematic calibration of the iCub humanoid robot. We have implemented a sequential Monte Carlo algorithm estimating kinematic calibration parameters (joint offsets) which improve the eye-hand coordination based on the proprioception and vision sensing of the robot. We have shown the usefulness of the developed code and its accuracy on simulation and real-world scenarios. The code is written in C++ and CUDA, where we exploit the GPU to increase the speed of the method. The code is made available online along with a Dataset for testing purposes.

## 1. Introduction and Related Work

An intelligent and autonomous robot must be robust to errors on its perceptual and motor systems to reach and grasp an object with great accuracy. The classical solution adopted by industrial robots rely on a precise calibration of the mechanics and sensing systems in controlled environments, where sub-millimeter accuracy can be achieved. However, a new emerging market is targeting consumer robots for collaboration with humans in more general scenarios. These robots cannot achieve high degrees of mechanical accuracy, due to (1) the use of lighter and flexible materials, compliant controllers for safe human-robot interaction, and (2) lower sensing precision due to varying environmental conditions. Indeed, humanoid robots, with complex kinematic chains, are among the most difficult platforms to calibrate and model properly with the precision required to reach and/or grasp objects. A small error in the beginning of the kinematic chain can generate a huge mismatch between the target location (usually coming from vision sensing) and the actual 6D end-effector pose.

Eye-hand calibration is a common problem in robotic systems that several authors tried to solve exploiting vision sensing [e.g., Gratal et al. (2011); Fanello et al. (2014); Garcia Cifuentes et al. (2017); Fantacci et al. (2017)]^1^.

## 2. Proposed Solution

In this code paper, we propose a markerless hand pose estimation software for the iCub humanoid robot [Metta et al. (2010)] along with an eye-hand kinematic calibration. We exploit the 3D CAD model of the robot embedded in a game engine, which works as the robot’s internal model. This tool is used to generate multiple hypotheses of the hand pose and compare them with the real visual perception. By using the information extracted from the robot motor encoders, we generate hypotheses of the hand pose and its appearance in the cameras, that are combined with the actual appearance of the hand in the real images, using particle filtering, a sequential Monte Carlo method. The best hypothesis of the 6D hand pose is used to estimate the corrective terms (joint offsets) to update the robot kinematic model. The visual based estimation of the hand pose is used as an input, together with the proprioception, to continuously calibrate (*i.e.*, update) the robot internal model. At the same time, the internal model is used to provide better hypotheses for the hand position in the camera images, therefore enhancing the robot perception. The two processes help each other, and the final outcome is that we can keep the internal model calibrated and obtain a good estimation of the hand pose, without using specialized visual markers on the hand.

The original research work [Vicente et al. (2016a) and Vicente et al. (2016b)] contains: (1) a complete motivation from the developmental psychology point of view and theoretical details of the estimation process, and (2) technical details on the interoperability between the several libraries and the GPGPU approach for an increased boost on the method speed, respectively.

The present manuscript is a companion and complementary code paper of the method presented in Vicente et al. (2016a). We will not describe with full details the theoretical perspective of our work, instead we will focus on the resulting software system connecting the code with the solution proposed in Vicente et al., 2016b. Moreover, the objective of this publication is to give a hands-on perspective on the implemented software which could be used and extended by the research community.

The source code is available at *the **official GitHub code repository***:

https://github.com/vicentepedro/Online-Body-Schema-Adaptation 

and the documentation on the ***Online Documentation page***:

http://vicentepedro.github.com/Online-Body-Schema-Adaptation

We use a Sequential Monte Carlo parameter estimation method to estimate the calibration error *β* in the 7D robot’s joint space corresponding to the kinematic chain going from each eye to the end-effector. Let us consider:

(1) $$\theta =\theta^{r}+\beta$$

where *θ^r^* are the real angles; *θ* are the measured angles; *β* are joint offsets representing calibration errors. Given an estimate of the joint offsets ($\hat{\beta }$), a better end-effector’s pose can be retrieved using the forward kinematics.

One of the proposed solutions for using Sequential Monte Carlo methods for parameter estimation^2^ (*i.e*., the parameters *β* in our problem), is to introduce an artificial dynamics, changing from a static transition model $\left(\beta_{t}=\beta_{t-1}\right)$ to a slowly time-varying one:

(2) $$\beta_{t}=\beta_{t-1}+{\text{w}}_{t}$$

where w*_t_* is an artificial dynamic noise that decreases when *t* increases.

## 3. Software Design and Architecture Principles

The software design and architecture for implementing the eye-hand kinematic calibration solution has the following requirements: (1) the software should be able to run in real-time since the objective is to calibrate the robot during a normal operating behaviour, and (2) it should be possible to run the algorithm in a distributed way, *i.e.*, run parts of the algorithm in several computers in order to increase computation power.

The authors decided to implement the code in C++ in order to cope with the real-time constraint, and to exploit the YARP middleware [Metta et al. (2006)] to distribute the components of the algorithm in more than one machine.

The source code for these modules are available at *the **official GitHub code repository*** (check section 2).

The code is divided into three logical components: (1) the hand pose estimation (section 4.1), (2) the Robot’s Internal Model generator (section 4.2), and (3) the likelihood assessment (section 4.3), which are implemented, respectively, at the following repository locations:

- modules/handPoseEstimation
  - include/handPoseEstimationModule.h
  - src/handPoseEstimationMain.cpp
  - src/handPoseEstimationModule.cpp
- modules/internalmodel
  - icub-internalmodel-rightA-cam-Lisbon.exe
  - icub-internalmodel-leftA-cam-Lisbon.exe
- modules/likelihodAssessment
  - src/Cuda_Gl.cu
  - src/likelihood.cpp

**Figure 1:**
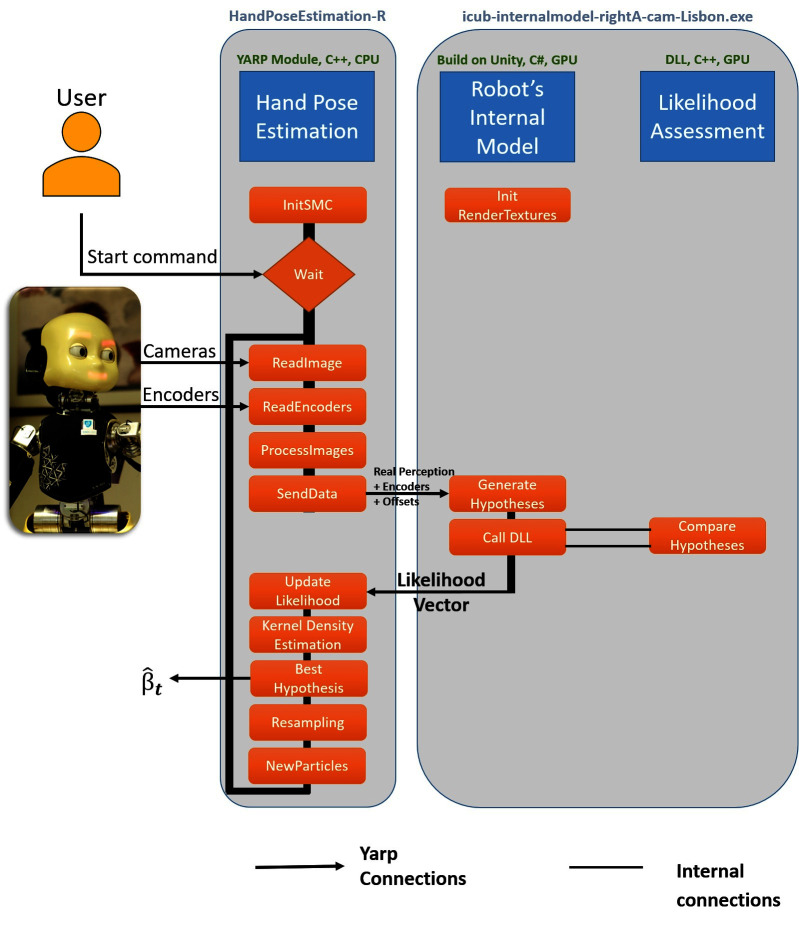
Architecture of the software. The hand pose estimation component (*handPoseEstimation*) initiates the Sequential Monte Carlo parameter estimation method (*initSMC*) and waits for a start command from the user. The perception and proprioception (cameras and encoders) of the robot are received and the parameter estimation starts. The real image and the particles are sent (*sendData*) to the Robot’s internal Model (*icub-internalmodel-rightA-cam-Lisbon.exe* or* icub-internalmodel-leftA-cam-Lisbon.exe*) in order to generate the hypotheses. The likelihood assessment of each hypothesis is calculated using a Dynamic Link Library (DLL) file inside the Robot’s internal model. The likelihood of each particle is saved and a Kernel Density estimation is performed to calculate the best calibration parameters. The Resampling step is performed and a new set of particles are saved for the next iteration of the Sequential Monte Carlo parameters estimation.

The software architecture implementing the proposed eye-hand calibration solution can be seen in Figure 1. The first component - Hand Pose Estimation - is responsible for proposing multiple hypotheses according to the posterior distribution. We use a Sequential Monte Carlo parameter estimation method in our work [check Vicente et al. (2016a) Section 3.3 for further theoretical details]. The definitions of the functions presented in the architecture (Figure 1) can be found in the .cpp and .h files and will be explained in detail in Section 4.1. The Hand Pose Estimation is OS independent and can run in any computer with the YARP library installed.

The second component - Robot’s Internal Model - generates hypotheses of the hand pose based on the 3D CAD model of the robot and was build using the game engine Unity^®^. There are two versions of the internal model on the repository. One for the right-hand (rightA) and another one for the left-hand (leftA). Our approach was to divide the two internal models since we have separated calibration parameters for the head-left-arm and for the head-right-hand kinematic chains. The Unity platform was chosen to develop the internal model of the robot since it is able to generate a high number of *frames per second* on the GPU even for complex graphics models. The scripting component of the Unity game engine was programmed in C#. The bindings of YARP for C# were used in order to facilitate the internal model generator to communicate with the other components of the system. This component is OS-dependent and only runs on Windows and the build version available on the repository does not require a paid license of Unity Pro.

Finally, the likelihood assessment is called inside the Robot’s Internal Model as a Dynamic Link Library and exploits GPGPU programming to compare the real perception with the multiple generated hypotheses. The GPGPU programming, using the CUDA library [Nickolls et al. (2008)], allows the algorithm to run in quasi-real-time. The .cpp file contains the likelihood computation method, and the .cu the GPGPU program.

Our eye-hand calibration solution exploits vision sensing to reduce the error between the perception and the simulated hypotheses, the OpenCV library [Bradski (2000)] with CUDA enabled capabilities [Nickolls et al. (2008)] was chosen to exploit computer vision algorithms and run them in real-time.

The interoperability between the OpenCV, CUDA and OpenGL libraries was studied in Vicente et al. (2016b). In the particular case of the iCub humanoid robot [Metta et al. (2010)], and to suit within the YARP and iCub architectures, we encapsulated part of the code in an RFModule^3^ class structure and use YARP buffered ports^4^ and RPC services^5^ for communications and user interface (Check section 5.2.3). The hand pose estimation module allows the user to send requests to the algorithm which follows an event-driven architecture: where for each new incoming information from the robot (cameras and encoders) a new iteration of the Sequential Monte Carlo parameter estimation is performed.

## 4. Code Description

### 4.1. Hand Pose Estimation Module

#### 4.1.1. Initializing the Sequential Monte Carlo parameter estimation - initSMC Function

In the function *initSMC* we initialize the variables of the Sequential Monte Carlo parameter estimation, *i.e.*, the initial distribution *p*(*β*_0_) [Eq. (10) in Vicente et al. (2016a)], and the initial artificial dynamic noise. The Listings 1 contains the *initSMC* function where some of the variables (in red) are parametrized at initialization time (check sub-section 5.2.1 for more details on the initialization parameters). We use a random seed generated according with the current time and initialize each angular offset with a Gaussian distribution: *N*(initialMean; initialStdDev).

Listing 1  **HandPoseEstimationModule::initSMC Function. Defined in handPoseEstimationModule.cpp**

1. bool handPoseEstimationModule :: initSMC ( )

2. {

4.  // Generate random particles

5.  srand((unsigned int)time(0)); // make sure random numbers are really random.

6.  rngState = cvRNG(rand());

7.  // initialize Beta1

8.  cvRandArr(&rngState, particles 1, CV_RAND_NORMAL, cvScalar(**initialMean**), cvScalar(**initialStdDev**));

9.  … … // similar for particles2 to particles6

10.  cvRandArr (&rngState, particles7, CV_RAND_NORMAL, cvScalar(**initialMean**) , cvScalar(**initialStdDev**));

11.  // Artificial Noise Initialization

12.  artifNoiseStdDev = **initialArtificialNoiseStdDev**;

13. }

#### 4.1.2 Read Image, Read Encoders, ProcessImages and SendData

The left and right images along with the head and arm encoders are read at the same time to ensure consistency between the several sensors.

The reading and processing procedure of the images are defined inside the function:

handPoseEstimationModule::updateModule() 

that can be found on the file: 

src/handPoseEstimationModule.cpp.

The function process Images (see Listings 2) applies a Canny edge detector and a distance transform to both images separately. Moreover, the left and the right processed images are merged, *i.e.*, concatenated horizontally, in order to be compared to the generated hypotheses inside the Robot’s internal model.

Listing 2  **HandPoseEstimationModule::processImages. Defined in handPoseEstimationModule.cpp**

1. Mat handPoseEstimationModule :: processImages (Mat inputImage)

2. {

3.  Mat edges , dt Image;

4.  cvtColor(inputImage, edges, CV_RGB2GRAY);

5.  // Blur Image

6.  blur(edges, edges, Size (3, 3));

7.  Canny(edges, edges, 65, 3*65,3);

8.  threshold(edges, edges, 100,255,THRESH_BINARY_INV); // binary Image

9.  distanceTransform(edges, dt Image, CV_DIST_L2, CV_DIST_MASK_5);

10.  return dtImage;

11. }

The Hand pose estimation module sends: (1) the pre-processed images, (2) the head encoders and (3) the arm encoders (*θ*) along with the offsets (*β*) to the Robot’s internal model^6^. This procedure is defined inside the function: 

handPoseEstimationModule::runSMCIteration()

#### 4.1.3. Update Likelihood

The Hand Pose Estimation module receives the likelihood vector from the Robot’s internal model and updates the likelihood value for each particle on the for-loop at line:

handPoseEstimationModule.cpp#L225

#### 4.1.4. Kernel Density Estimation

Although the state is represented at each time step as a distribution approximated by the weighted particles, our best guess for the angular offsets can be computed using a Kernel Density Estimation (KDE) to smooth the weight of the particles according to the information of neighbor particles, and choose the particle with the highest smoothed weight (*ω*ʹ^[*i*]^) as our state estimate [Section 3.5 of Vicente et al. (2016a)].

The implementation of the KDE with a Gaussian kernel can be seen in Listings 3. The double for-loop implements the KDE accessing each particle (*iParticle*) and computing the influence of each neighbor (*mParticle*) according to the relative distance in the 7D-space between the two particles and the likelihood of the neighbor *[cvmGet (particles, 7,mParticle)*]. The parameters that can be fine-tuned are highlighted in red.

Listing 3  **Kernel Density Estimation with Multivariate Normal Distribution Kernel: modules/handPoseEstimation/src/handPoseEstimationModule.cpp**

1. void handPoseEstimationModule :: kernelDensityEstimation ( )

2.{

3. // Particle i 

4. double maxWeight = 0.0;

5. for (int iParticle = 0; iParticle <n Particles; iParticle ++) 

6. {

7.  double sum1 = 0.0;

8.  // Particle m

9.   for (int mParticle = 0; mParticle <nParticles; mParticle++)

10.  {

11.   double sum2 = 0.0;

12.   if ( (float) cvmGet (particles, 7, mParticle) > 0 )

13.   {

14.    // Beta 0.. to..6

15.    for (int joint = 0; joint <7; joint ++)

16.    {

17.     // || pi–pj ||^^^2 / KDEStdDev ^^^2

18.     sum2 += pow( ((float) cvmGet (particles, joint, mParticle)–(float)  cvmGet (particles, joint, iParticle )) , 2) / pow(**KDEStdDev**, 2); //  Multivariate normal distribution

19.    }

20.    sum1 += s t d :: exp(–sum2/( 2) ) *cvmGet (particles, 7 ,  mParticle);

21.   }

22.  }

23.  sum1 = sum1 / ( nParticles*sqrt (pow(2*M_PI, 1) *pow(**KDEStdDev**,  7) ) ); 

24.  double weight = alphaKDE*sum1 + cvmGet (particles, 7 ,  iParticle); 

25.  if (weight>maxWeight)

26.  {

27.   maxWeightIndex= iParticle; // save the best particle index

28.  }

29. }

30.}

#### 4.1.5. Best Hypothesis

The best hypothesis, computed using the KDE, is sent through a YARP buffered port from the module after *N* iterations. The port has the following name:

/hpe/bestOffsets:o

The parameter *N* (the number of elapsed iterations before sending the estimated angular offsets) can be changed by the user at initialization using the minIteration parameter (check Section 5.2.1 for more details) and the objective is to ensure the filter convergence before using the estimate (*e.g.*, to control the robot). This is an important parameter since in the initial stages the estimation can jump a lot from an iteration to the next one (before converging to a more stable solution).

#### 4.1.6. Update Artificial Noise, Resampling and New Particles

The artificial noise is updated according to the maximum likelihood criteria. See the pseudo-code on Listings 4, which corresponds to line 230 to 254 in the file:

src/handPoseEstimationModule.cpp

Listing 4  **Pseudo Code updating artificial noise corresponding to part of the function runSMCIteration() within file: *src/handPoseEstimationModule.cpp***

1. IN handPoseEstimationModule :: runSMCIteration ( )

2.{

3. …

4. // Resampling or not Resampling. That’s the Question

5. if (maxLikelihood >**minimumLikelihood**) {

6.  systematic_resampling ( ); // Check Section Resampling and New Particles

7.  reduceArtificialNoise ( );

8. }

9. else { // do not apply resampling stage

10.  increaseArtificialNoise ( );

11. }

12. if (artifNoiseStdDev > **upperBoundNoise**) { // upperbound of artificial noise

13.  artifNoiseStdDev = **upperBoundNoise**;

14. }

15. if (artifNoiseStdDev < **lowerBoundNoise**) { // lowerbound of artificial noise

16.  artifNoiseStdDev = **lowerBoundNoise**;

17. }

18. addNoiseToEachSample ()

19.}

We update the artificial noise according to the maximum likelihood, *i.e.*, if the maximum likelihood is below a certain threshold (*minimumLikelihood*), we do not perform the resampling step and we increase the artificial noise. On the other hand, if the maximum likelihood is greater than the threshold we apply the resampling and decrease the artificial noise. The objective is to prevent the particles to become trapped in a “local maximum” since the current best solution is not worthy of resampling the particles. Indeed, this approach will force them to explore the state space.

The trade-off between exploration and exploitation is measured according to the maximum likelihood in each time step of the algorithm. The idea is to exploit the low number of particles in a clever way. Moreover, the upper and lower bound ensure, respectively, that: (1) the noise will not increase asymptotically and the samples will be spread over the 7D state-space and (2) the particles will not end-up all at the same value, which can happen when the random noise is Zero.

On the resampling stage, we use the systematic resampling strategy [check Hol et al. (2006)], which ensures that a particle with a weight greater than 1/*M* is always resampled, where *M* is the number of particles.

### 4.2. Robot’s Internal Model Generator

The Listings 5 shows the general architecture of the Robot’s Internal Model Generator using pseudo-code.

Listing 5  **Pseudo-Code Robot's internal model.**

1. InitRenderTextures ( ) // Initialization of the strutures to receive

2.

3.for (each iteration) // for each iteration of the SMC

4.{

5. waitForInput ( ); // wait for input vector with particles to be generated

6.

7. for (each particle) {

8.  moveTheInternalModel ( ) // Change the robot’s configuration

9.  RenderAllucinatedImages ( ); // render left and right image on a render texture

10.  nextFrame ( );

11. }

12. // After 200 frames call DLL function

13. ComputeLikelihood (AllucinatedImages (200), RealImage) // Call the DLL function (CudaEdgeLikelihood) to compare the hypotheses with the real image.

14.}

#### 4.2.1. Initialization of the Render Textures

The render textures, which will be used to render the two camera images, are initialized for each particle for both left and right views of the scene.

#### 4.2.2. Generate Hypotheses

The hypotheses are generated on a frame-based approach, *i.e.*, we generate one hypothesis for each frame of the “game”. After we receive the vector with the 200 hypotheses to generate, we virtually move the robot to each of the configurations to be tested and record both images (left and right) in a renderTexture.

After the 200 generations, we call the likelihood assessment DLL function to perform the comparison between the real images and the generated hypotheses.

The available version of the Robot’s internal model generator is an executable compiled and self-contained which works on Windows-based computers with the installed dependencies^7^. Moreover, this does not require neither the Unity^®^ Editor to be installed in the computer nor the Unity Pro license.

More details on the creation of the Unity^®^ iCub Simulator for this project can be found in Vicente et al. (2016b) Sec. 5.2 - “The Unity^®^ iCub Simulator”.

### 4.3. Likelihood Assessment Module

The likelihood assessment is based on the observation model defined in Vicente et al. (2016a) Section 3.4.2.

We exploit an edge-based extraction approach along with a distance transform algorithm computing the likelihood using the Chamfer matching distance [Borgefors and Bradski (1986)].

In our code, these quantities are computed in the GPU using the OpenCV and CUDA libraries, and the interoperability between these libraries and the OpenGL library. The solution adopted was to add the likelihood assessment as a cpp plugin called inside the internal model generator module. The likelihood.cpp file, particularly the function *CudaEdgeLikelihood*, is where the likelihood of each sample is computed. Part of the code of the likelihood function is shown and analysed in Listings 6. Up to the line 21 of the Listings 6, we exploit the interoperability between the libraries used (OpenGL, CUDA, OpenCV) and after line 21 we apply our likelihood metric using the functionality of the OpenCV library, where *GgpuMat* is the generated Image of the *ith* sample and GgpuMat_R is the real Distance Transform image. In line 35, the lambdaEdge is a parameter to tune the distance metric sensitivity, which is initialized at the value 25 in line 1 (corresponding to line 148 of the C++ file)^8^. When the generated image does not have edges (*i.e.*, the hand is not visible by the cameras), we force the likelihood of this particle to be almost zero (line 37 and 39, respectively). The maximum likelihood (*i.e.*, the value 1.0) is achieved when each entry of the result image is zero. This happen when every edge on the generated image match a zero distance on the distance transform image. The multiplication by 1,000 and the *int* cast in line 42 is used to send the likelihood as a int value (the inverse process is made in the internal model when it receives the likelihood vector) and it is one of the limitations of the current approach due to software limitations the authors could not send directly a double value between 0 and 1.

Listing 6  **Likelihood Assessment: modules/likelihoodAssessment/src/likelihood.cpp**

1. int lambdaEdge = 25;

2. // For each particle i – line 149 modules / likelihoodAssessment / src / likelihood.cpp

3. // Interopelability between the several libraries (OpenGL , CUDA, OpenCV)

4. gltex =(GLuint) (size_t) (ID[i]); // ID is a vector with pointers to the render textures

5. glBindTexture(GL_TEXTURE_2D, gltex);

6. GLint width, height, internalFormat;

7. glGetTexLevelParameteriv(GLTEXTURE_2D, 0, GL_TEXTURE_COMPONENTS, &internalFormat); // get internal format type of GL texture

8. glGetTexLevelParameteriv(GL_TEXTURE_2D, 0, GL_TEXTURE_WIDTH, &width); // get width of GL texture

9. glGetTexLevelParameteriv(GL_TEXTURE_2D, 0, GL_TEXTURE_HEIGHT, &height); // get height of GL texture

10.

11. checkCudaErrors( cudaGraphicsGLRegisterImage ( &cuda_tex_screen_resource , gltex , GL_TEXTURE_2D, cudaGraphicsMapFlagsReadOnly ) );

12. // Copy color buffer

13. checkCudaErrors( cudaGraphicsMapResources ( 1, &cuda_tex_screen_resource , 0 ) );

14. checkCudaErrors( cudaGraphicsSubResourceGe tMappedArray ( &cuArr , cuda_tex_screen_resource, 0, 0 ) );

15. BindToTexture( cuArr); // BindToTexture Functions defined in Cuda_Gl.cu

16.

17. DeviceArrayCopyFromTexture( ( float3*) gpuMat.data, gpuMat.step, gpuMat.cols, gpuMat.rows );//DeviceArrayCopyFromTexture function defined on Cuda_Gl.cu

18.

19. checkCudaErrors( cudaGraphicsUnmapResources ( 1, &cuda_tex_screen_resource, 0 ) );

20. checkCudaErrors( cudaGraphicsUnregisterResource (cuda_tex_screen_resource) );

21. cv::gpu::cvtColor(gpuMat, GgpuMat,CV_RGB2GRAY);

22.

23. // Apply the likelihood Assessment

24. // GgpuMat – generated Image

25. // GgpuMat_R – Real Distance Transform image

26. cv :: gpu :: multiply (GgpuMat, GgpuMat_R, GpuMatMul);

27. cv :: Scalar sumS = cv :: gpu :: sum(GpuMatMul);

28.

29. /* 

30. Check the article:

31. Online Body Schema Adaptation Based on Internal Mental Simulation and Multisensory Feedback, Vicente et al.

32. In particular, Equation (21)

34. */

35. sum = sumS [0]*lambdaEdge; // lambdaEdge is a tuning parameter for distance sensitivity

36. nonZero = (float) cv::gpu::countNonZero (GgpuMat); // generated image

37. if (nonZero ==0) {

38.  likelihood [i] = 0.000000001; // Almost Zero

39. }

40. else {

41.  result = sum/nonZero;

42.  likelihood[i] = (int) ((cv::exp(– result)) *1000);

43. }

44.}

## 5. Application and Utility

The Markerless kinematic calibration can run during normal operations of the iCub robot. It will update the joint offsets according to the new incoming observations. Moreover, one can also stop the calibration and use the estimated offsets so far, however, to achieve a better accuracy in different poses of the end-effector the method should be kept running in an online fashion to perform a better adaptation of the parameters.

The details of the dependencies, installation and how to run the modules can be found at ***Online Documentation page*** (check Section 2).

### 5.1. Installation and Dependencies

The dependencies of the proposed solution can be divided in two sets of libraries: (1) the libraries needed to run the handPoseEstimation module, and (2) the libraries needed to run the Robot’s internal model and the likelihood Assessment.

#### 5.1.1. Hand Pose Estimation Module

The handPoseEstimation depends on YARP library, which can be installed following the installation procedure of the official repository^9^. Moreover, it depends on the OpenCV library^10^.

We tested this module with the last release of YARP (*i.e.*, June 15, 2017), version 2.3.70, with the OpenCV library V2.4.10 and V3.3 and the code works with both versions. The authors recommend the reader to follow the official installation guides for these libraries.

To install theses modules, one can just run *CMake* using the *CMakeLists.txt* on the folder:

/modules/handPoseEstimation/

#### 5.1.2 Robot’s Internal Model Generator and Likelihood Assessment

The Robot’s internal model and the likelihood assessment depend on YARP library for communication and on the OpenCV library with CUDA enabled computation (*i.e.*, installing the CUDA toolkit) for image processing and GPGPU accelerated algorithms. A Windows machine should be used to install this module.

The tested version of the OpenCV library was V.2.4.10 with the CUDA toolkit 6.5. The C# bindings for the YARP middleware on a windows machine should be compiled. The details regarding the installations procedures can be found at the following URL: http://www.yarp.it/yarp_swig.html#yarp_swig_windows.

The C# bindings will allow the internal model generator to communicate with the other modules.

The C# bindings will generate a DLL file that, along with the DLL generated from the likelihood assessment module, should be copied to the Plugins folder of the internal model generator. In the official compiled version of the repository this folder has the following path: internalmodel/icub-internalmodel-rightA-cam-Lisbon_Data/Plugins/

The complete and step-by-step installation procedure can be seen in the ***Online Documentation page*** on the Installation section.

### 5.2. Running the Modules

The proposed method can run on a cluster of computers connected with the YARP middleware. The internal model generator should run on a computer with Windows Operating System and with CUDA capabilities. The step-by-step running procedure guide can be found on the***Online Documentation page***. The rest of the section is organized with a high level perspective of running the algorithm. The YARP connections required between the several components can be connected through the XML file under the app/scripts folder.

#### 5.2.1. Running the Hand Pose Estimation and its parameters

The Hand Pose Estimation can be initialized using the yarpmanager or in a terminal running the command: 

handPoseEstimation [--<parameter_name> <value > …] 

where, <value> is the value for one of the parameters (<parameter_name>) defined in the itemize list below:

- name: name of the module (default =“hpe”)
- arm: arm which the module should connect to. (default = right’)
- initialMean: mean for the initial distribution of the particles [in degrees]. (default = 0.0°)
- initialStdDev: StdDev of the initial distribution of the particles degrees
- artificialNoiseStdDev: initial Artificial Noise (StdDev) to spread the particles after each iteration (default = 3.0°)
- lowerBound: artificial noise lower bound (StdDev). Should be greater than Zero to prevent the particles to collapse in one single value (default = 0.04°)
- upperBound: artificial noise upper bound (StdDev). The artificial noise should have a upper bound to prevent the particles to diverge after each resampling stage (default = 3.5°)
- minimumLikelihood: minimumLikelihood [0,1] in order to resample the particles (default = 0.55)
- increaseMultiplier: increase the artificial noise of a certain value (currentValue*increaseMultiplier) if the maximum likelihood is lower than the minimumLikelihood (default = 1.15)
- decreaseMultiplier: decrease the artificial noise of a certain value (currentValue*decreaseMultiplier) if the maximum likelihood is greater than the minimumLikelihood (default = 0.85)
- KDEStdDev: StdDev of each kernel in the Kernel Density Estimation algorithm (default = 1.0°)
- minIteration: minimum number of iterations before sending the estimated offsets. The objective is to give time to the algorithm to converge, without this feature one can receive completely different offsets from iteration *t* to *t* + 1 during the filter convergence (default = 35)

#### 5.2.2. Running the Robot’s Internal Model

The internal model generator should run on a terminal using the following command:

icub-internalmodel-rightA-cam-Lisbon.exe -force-opengl

The -force-opengl argument will force the robot’s internal model to use the OpenGL library for rendering purposes, which is fundamental for the libraries interoperability.

#### 5.2.3. User interface

The user can send commands to the Hand Pose estimation algorithm through the RPC port *hpe/rpc:i*. The RPC port acts like a service to the user where the algorithm can be started, stoped or paused/resumed. It is also possible to request the last joint offsets estimated by the algorithm. The thrift file (*modules/handPoseEstimation/handPoseEstimation.thrift*) contains the input and output of each RPC service function (*i.e.*,* start, stop, pause, resume, lastOffsets and quit*). More details about these commands can be seen in the use procedure on the documentation. Moreover, after connecting to the RPC port (*yarp rpc hpe/rpc:i*), the user can type help to get the available commands. The module also replies the input and output parameters of a given command if the user type *help FunctionName* (*e.g., help start*).

## 6. Experiments and Examples of Use

The experiments performed with the proposed method on the iCub simulator, with ground truth data, have shown a good accuracy on the hand pose estimation, where artificial offsets were introduced in the seven joints of the arm. The results on the real robot have shown a significant reduction of the calibration error [Check Vicente et al. (2016a) Section 5 for more results in simulation (Section 5.1) and with the real iCub (Section 5.2)].

For the reader to be able to test the algorithm, the authors collected a simulated dataset (encoders of the head and arms, and the left and right images) which can be used to test the algorithm. The simulation results of the present article were obtained running the above-stated code with the default parameters on the collected dataset.

The dataset^11^ was collected using a visual simulator based on the CAD model of the iCub humanoid robot adding artificial offsets in the arm joints. The artificial angular offsets *β* were the following:

*β* = { – 10.0, – 10.0, 6.0, – 7.0, – 1.0, – 20.0, 7.0}°.

**Figure 2:**
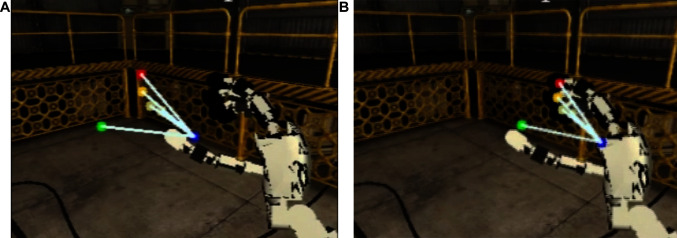
Projection of the fingertips on the left camera on simulated robot experiments. The blue dot represents the end-effector projection (*i.e.*, base of the middle finger), the red represents the index fingertip, the green the thumb fingertip, the dark yellow the middle fingertip and the soft yellow the ring and little fingertips. On the left image **(A)** is the canonical projection (*i.e.*, with $\hat{\beta }=0$) and on the right image **(B)** the estimated offsets ($\hat{\beta }$).

The robot performed a babbling movement which consists in a random walk in each joint. The minimum and maximum values of the uniform distribution used to generate the babbling movement starts at [–5, 5]°, and is reduced during the movement to [– 0.5, 0.5]°, respectively. Despite a great amount of errors in the robot’s kinematic chain, the algorithm was able to converge to the solution in Figure 2. Moreover, the cluttered environment on the background did not influence the filter convergence. The reader can see the projection of the fingertips on the left camera image: (1) according to the canonical representation on Figure 2A (where it is assumed an error-free kinematic structure, *i.e.*, with $\hat{\beta }=0$ and (2) the corrected kinematic structure using the algorithm implemented and documented in this code paper on Figure 2B.

The convergence of the algorithm along with a side-by-side comparison with the canonical solution can be seen in the following video: https://youtu.be/0tzLFqZLbxc

**Figure 3:**
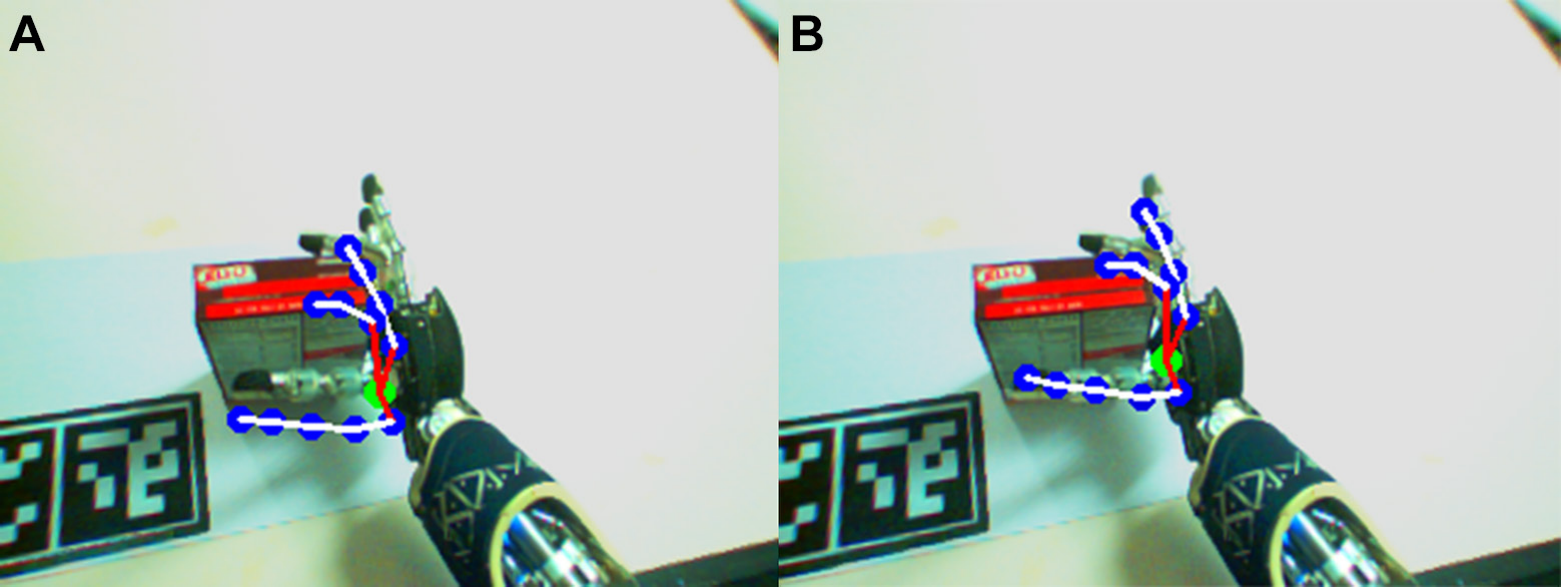
Projection of the fingertips on left camera in real robot experiments. On the left image **(A)** the canonical projection (*i.e.*, with $\hat{\beta }=0$) is shown, and on the right **(B)** the projection according with the corrected kinematic chain using the estimated offsets ($\hat{\beta }$).

On the real robot, we already performed several experiments in previous works, with different initial and final poses using the 320 × 240 cameras. In Figure 3 one can see one example of the hand estimation. While the image on the left (Figure 3A) shows the canonical estimation of the hand projected on the left camera image according to the non-calibrated kinematic chain, the image on the right (Figure 3B) shows the corrected kinematic chain which originates a better estimation of the hand pose. The rendering of the estimated hand pose was done taking into account the joint offsets on the kinematic chain before computing the hand pose in the image reference frame.

## 7. Known Issues

There are some known issues or limitations in this algorithm and its software. The Windows dependency of the internal model generator module can be a problem for non-windows users. Moreover, the number of particles in the Sequential Monte Carlo is fixed (200 particles), which we found to be a good trade-off between accuracy and speed [check Vicente et al. (2016a) for more details on this matter].

The camera size is also fixed to the 320 × 240 resolution, which is sufficient to most of the experiments performed on the iCub. Indeed, to the authors’ knowledge, this is the most popular resolution in the iCub community. The camera resolution can be modified by changing the input resolution on the hand pose estimation module and on the internal structures of the internal model and the likelihood assessment. However, this demands for a recompilation of the internal model generator which could not be done without a Pro license of Unity^®^.

The limitation on the integration of the likelihood assessment and the *int* cast discussed in Section 4.3 should be investigated since we are truncating the likelihood and in the end we have, at most, three significant figures of the likelihood value.

Hand occlusions can also be problematic at this stage of the work since we are not dealing explicitly with them. If the hand is occluded for a long period, the filter can start to diverge since it does not find a good match of the hand model in its perception.

## 8. Conclusion and Future Work

In this paper, we have shown how to calibrate the eye-hand kinematic chain of a humanoid robot – the iCub robot. We have provided a tutorial on how to execute the module and how it works, its inputs and outputs. Our proposed work could be beneficial for research works with the iCub humanoid robot, from manipulation related fields to human-robot interaction, for instance. The results have shown a good accuracy in simulation and in a real-world environment. For future work, we are planning to extend the architecture. A useful feature is to be able to predict if the hand is present or not in the image or if it is occluded in order to perform a better match between the perception and the generated hypotheses. We will investigate the possibility of running the internal model simulator on different platforms (*i.e.*, Linux, macOS), which seems to be a new feature of the Unity game engine editor environment.

## Author Contributions

In this work, all the authors contributed to the conception of the markerless eye-hand kinematic calibration solution and to the analysis and interpretation of the data acquired.

## Conflict of Interest Statement

The authors declare that the research was conducted in the absence of any commercial or financial relationships that could be construed as a potential conflict of interest.

The reviewer, CF, and handling Editor declared their shared affiliation.

## Footnotes

1 For a more detailed review of the state of the art, please check the article Vicente et al. (2016a)

2 See Kantas et al. (2009) for other solutions

3 http://www.yarp.it/classyarp_1_1os_1_1RFModule.html

4 http://www.yarp.it/classyarp_1_1os_1_1BufferedPort.html

5 http://www.yarp.it/classyarp_1_1os_1_1RpcServer.html

6 See Eq. 1 and handPoseEstimationModule.cpp#L214

7 The list of dependencies can be seen on Section 5.1.2

8 Check Vicente et al. (2016a) Eq (21) for more details on the lambdaEdge parameter

9 https://github.com/robotology/yarp

10 It is not mandatory the CUDA-enabled capabilities

11 https://github.com/vicentepedro/eyeHandCalibrationDataset-Sim
